# Residents’ Waste Separation Behaviors at the Source: Using SEM with the Theory of Planned Behavior in Guangzhou, China

**DOI:** 10.3390/ijerph120809475

**Published:** 2015-08-12

**Authors:** Dongliang Zhang, Guangqing Huang, Xiaoling Yin, Qinghua Gong

**Affiliations:** 1 Guangzhou Institute of Geochemistry, Chinese Academy of Sciences, Guangzhou 510640, China; 2 Guangzhou Institute of Geography, Guangzhou 510070, China; E-Mails: hgq@gdas.ac.cn (G.H.); yinxl@gdas.ac.cn (X.Y.); gqh100608@163.com (Q.G.); 3 University of Chinese Academy of Sciences, Beijing 100049, China

**Keywords:** theory of planned behavior, waste separation behavior, structural equation modeling, environmental education, China

## Abstract

Understanding the factors that affect residents’ waste separation behaviors helps in constructing effective environmental campaigns for a community. Using the theory of planned behavior (TPB), this study examines factors associated with waste separation behaviors by analyzing responses to questionnaires distributed in Guangzhou, China. Data drawn from 208 of 1000-field questionnaires were used to assess socio-demographic factors and the TPB constructs (*i.e.*, attitudes, subjective norms, perceived behavioral control, intentions, and situational factors). The questionnaire data revealed that attitudes, subjective norms, perceived behavioral control, intentions, and situational factors significantly predicted household waste behaviors in Guangzhou, China. Through a structural equation modeling analysis, we concluded that campaigns targeting moral obligations may be particularly effective for increasing the participation rate in waste separation behaviors.

## 1. Introduction

Household solid waste (HSW) management has been and will continue to be a major issue facing countries worldwide [1], particularly in the cities of developing countries [2]. HSW is generally defined as waste produced by normal household activities, and it is a major source of municipal solid waste [3]. China’s continuing development, with its current total population of approximately 1.35 billion and its industrialization and urbanization, will accelerate the daily generation and volume rate of HSW. With a total HSW generation of 170.81 million tons in 2012, the average generation rate was 0.35 kg/capita/day [4]. The waste management sector were faced with numerous challenges, such as a land shortage for landfills and residents opposed to waste disposal facilities constructed near their homes. One way to overcome these problems is with recycling, and the important prerequisite for recycling is separation of HSW at the source. The successful management and marketing of any HSW recycling scheme will require national and local governments to encourage high levels of public participation to ensure that the planned technology is implemented successfully [5].

The theory of planned behavior (TPB) provides a theoretical framework for systematically identifying the factors that influence waste separation. Several empirical studies have found factors that influence waste handling behaviors based on TPB. Chan [6] interviewed 173 household members in a public housing estate in Hong Kong. He found that attitude was the major factor predicting a pro-environmental behavioral intention and that publicity messages from the mass media should be effective for promoting green behaviors. Tonglet *et al.* [7] concluded that pro-recycling attitudes are the major contributors to recycling behavior and found that such attitudes were influenced mainly by acquiring the appropriate opportunities, facilities, and knowledge regarding recycle. Karim *et al.* [5] concluded that people’s attitudes toward waste separation were the main predictors of forming waste separation intentions. Other similar studies [8,9,10] have found attitudes to be the main predictors concerning behavioral intentions. In Mahmud’s [11] study, perceived behavioral control was the strongest predictor of intentional behavior, and the specific attitudes were indirect predictors. Similarly, Bortoleto *et al.* [12] concluded that personal norms and perceived behavioral control were the main predictors of household waste prevention behavior.

In sum, this review of some of the recent literature on waste handling behaviors based on TPB suggests that attitudes are the main predictors of behavioral intentions. Almost all of these studies were conducted in developed economies, such as the U.S. and in Europe, but similar studies in developing countries are scarce, except for Malaysia. Given this context, we conducted an investigation to identify and evaluate the factors that influence waste separation behaviors (WSB) in households in Guangzhou, China. If these factors can be identified, specific measures can be taken to successfully implement a source separation plan. The research questions framed for this investigation are as follows:

(a) What are the factors that significantly influence WSB?
(b) To what extent do these factors predict WSB?
(c) How strong are the relationships of these factors to WSB?

To answer these questions, we initially adopted a hypothetical model that was empirically applied to questionnaire data through structural equation modeling (SEM).

## 2. Theoretical Basis and Research Hypotheses

### 2.1. Theory of Planned Behavior (TPB)

**Figure 1:**
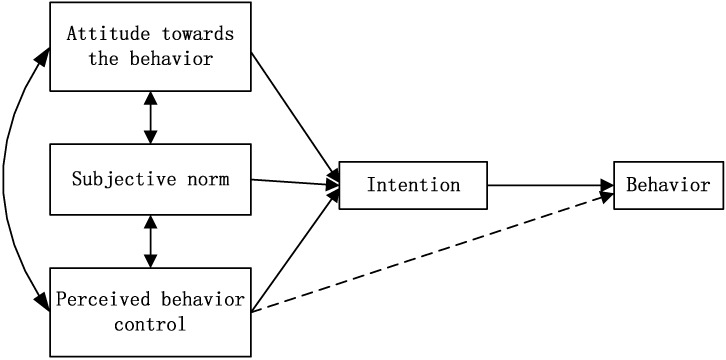
Schematic representation of the theory of planned behavior.

Source separation is the primary step in HSW management. There is a need for theory-based studies to better understand the mechanisms responsible for separation behaviors. Ajzen’s [13] theory of planned behavior (TPB) provides a theoretical framework for systematically examining behavior concerning waste separation. TPB has been widely used to investigate waste behaviors [5,12,14]. According to the theory (Figure 1), an individual’s behavior is based on his or her readiness to perform that behavior (*i.e.*, intention). Intention is based on three factors: (1) attitude (A), which is the individual’s positive or negative perception of performing a behavior; (2) subjective norm (SN), which is the individual’s perception of social pressure to engage or not in a behavior; and (3) perceived behavioral control (PCB), which is the individual’s perception of his or her ability to perform a given behavior.

Within the context of the TPB, more attention should be given to identifying the factors that influence separation behaviors. Several studies have recommended adding more variables to improve the predictive validity of the TPB. For example, moral obligation had a significant influence on prevention behaviors among households in São Paulo, Brazil [12]. Davis suggested that situational factors should also be included as a variable in the model [15]. This variable was measured by assessing the extent to which the respondents perceived situational factors, such as limited space, time, and inconvenience, as barriers to carrying out waste separation behavior. Ramayah added environmental knowledge to his model [9]. To our knowledge, no study has investigated household waste separation behaviors in China from the perspective of the TPB. The goal of this study was to identify and evaluate the influential factors on attitudes toward participation in source separation of waste among households in a community sample in China.

### 2.2. Research Hypotheses

**Figure 2:**
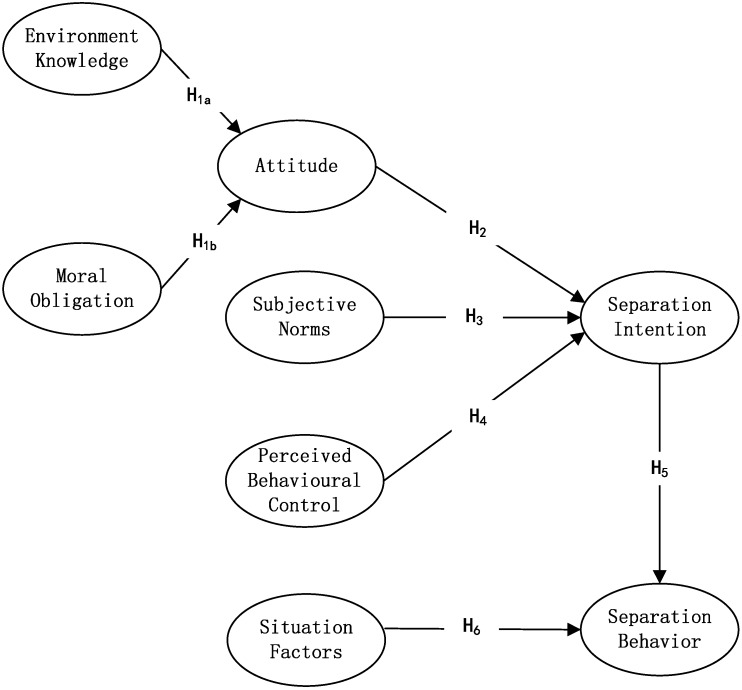
The hypothetical structural equation model of waste separation behavior.

Figure 2 shows the hypothetical structural equation model for WSB that we adopted, with its indicated hypotheses. The specific hypotheses regarding WSB are stated below.

**H1a.** Environmental knowledge is positively related to attitude.

The existence of a positive relationship between environmental knowledge and environmental behavior is supported by the results of previous studies [16,17,18]. In particular, many studies demonstrated that specific knowledge of a given behavioral scheme is significantly and positively related to individuals’ attitudes [19]. Environmentally knowledgeable people have more positive environmental attitudes in reality, as well as better environmental behavioral intentions.

**H1b.** Moral obligation has a positive influence on attitudes.

Barr *et al.* [20] described attitudes that stress a moral obligation toward the environment along with active individual beliefs that emphasize rights and responsibilities. Theoretically, a resident who has a high sense of moral obligation often regulates his or her thoughts and behaviors in life, thereby developing a better environmental attitude as well.

**H2.** An individual’s attitude toward WSB has a p effect on WSB intention.

One’s attitudes based on one’s perception of a behavior as positive or negative, right or wrong, pleasant or unpleasant, or interesting or boring. Karim *et al.* [5] found that personal attitude had the strongest correlation with waste separation intention. A positive attitude results in a positive belief in oneself, such as the belief that participation in WSB will reduce pollution and the wasteful use of landfills. The results has also been confirmed by Nigbur *et al.* [21], in a study of curbside recycling in the UK, found that attitude predicted the intention to recycle, which, in turn, predicted recycling behavior.

**H3.** Subjective norms have a positive influence on WSB intention.

Subjective norms are social factors that include perceived social pressures to engage or not in a certain behavior. Possible sources of these social factors include pressure from family, neighbors, peers, or the community. Some studies have found that people’s recycling behavior intentions are substantially influenced by the social norms that they perceive are held by other persons or social groups that are important to them [22,23]. Thus, social norms are positively related to a person’s WSB intention.

**H4.** Perceived behavioral control has a positive influence on WSB intention.

Perceived behavioral control reflects an individual’s past experience and anticipates obstacles. The more resources and opportunities a person perceives in performing a specific behavior and the fewer the expected obstacles, the stronger the perceived behavioral control, making the behavior more likely to occur. Many researchers have examined the relationship between perceived behavioral control and the intention to perform a behavior [12,14,24].

**H5.** An intention toward WSB has a positive effect on WSB.

A combination of attitude, subjective norms, and perceived behavioral control will result in the formation of a behavioral intention. It has been established that a combined impact of more favorable attitude and subjective norm together with greater perceived behavioral control lead to stronger intention to perform a given behavior. Moreover, the stronger the intentions, the greater the likelihood that people will behave according to these intentions. Pakpour [14] found that intention had a strong correlation with recycling behavior, whereas Karim *et al.* [5] concluded that the relationship between intention and behavior was significant and positive but small.

**H6.** Situational factors have a significant influence on WSB.

Situational factors are individuals’ objective environment when they perform a particular behavior. This variable is used to assess the extent to which respondents’ situational factors, such as limited space, time, and inconvenience, are barriers to performing waste separation behavior. Karim *et al.* [5] concluded that situational factors significantly influenced waste separation intentions.

## 3. Method

### 3.1. Study Area

Guangzhou is located on the southern Chinese mainland at 112°57′E～114°3′E, 22°26′N～23°56′N. Guangzhou has a subtropical marine monsoon climate, with an annual precipitation of about 1720 mm and annual average temperature of 20–22 °C. The city covers an area of 7434 km^2^ with a resident population of 12.84 million as of 2012 [25]. As a consequence of rapid urbanization, increasing population, and economic development, the total municipal solid waste (MSW) generated in Guangzhou increased from 2.31 million tons in 2003 to 4.13 million tons in 2012, with an average annual increase of 6.67%. Table 1 shows Guangzhou’s yearly growth in population, wages, generation intensity, and generated MSW between 2007 and 2013. Note that the population in Guangzhou increased from 10.05 million in 2007 to 12.93 million in 2013, with an average annual increase of 4.29%. Over the same period, the average wage increased faster than the population, with an average annual increase of 9.61%.

**Table 1:** Population, income, and total MSW generated in Guangzhou between 2007 and 2013.

Year	Population ^a^ (Millions)	Average Annual Income of Employed Staff and Workers ^a^ (CNY ^b^)	MSW Generated ^a^ (Million Tons)	Generation Intensity ^c^ (kg/capita/day)
2007	10.05	40187	3.40	0.93
2008	10.18	45365	3.17	0.85
2009	10.33	49215	3.71	0.98
2010	12.71	54495	3.57	0.77
2011	12.75	57473	3.49	0.75
2012	12.84	63752	3.80	0.81
2013	12.93	69692	3.94	0.83

**^a^** Guangzhou Statistical Yearbook, 2008–2013; **^b^** ISO Currency Code of the Chinese Yuan (CNY); **^c^** Calculated by the authors.

### 3.2. Questionnaire Design

The questionnaire for the present study was administered during July and August of 2014. It was designed to fulfill a number of key requirements as described in Section 2.2. The recipients received an envelope containing a cover letter and a two-page questionnaire consisting of structured questions relating to the hypothetical model. We set up questionnaire collection boxes in each residential building lobby and collected the completed questionnaires one week after they had been distributed.

The questionnaire had nine conceptual sections: (1) knowledge about waste separation issues, (2) moral obligations, (3) attitudes toward waste-specific issues, (4) subjective norms, (5) perceived behavioral control, (6) situational factors, (7) intentions of WSB, (8) WSB, and (9) demographic information. For each questionnaire item, excluding the questions on demographics, the respondents were asked to indicate the extent of their agreement with the given statements on 5-point Likert scales, where 1 = *strongly disagree* and 5 = *strongly agree*.

### 3.3. Measurement Instruments

Structural equation modeling (SEM) deals with theoretical models of complex phenomena by use of certain statistical analytical techniques (e.g., measurement theory, factor analysis, regression, and path analysis). To conduct quantitative research on practical problems, the SEM evaluates the theoretical model according to the extent of consistency between the theoretical model and the actual data. The essence of SEM is an extension of the general linear model, which combines factor analysis and path analysis. Variables in the SEM usually include observed variables that are directly measured and latent variables that cannot be measured. A latent variable cannot be seen; its presence is inferred from what is observed. In waste management research, it is impossible to observe each household and speculate about latent variables. Instead, a questionnaire distributed to individuals can measure indirectly the extent of these latent variables. Respondents were asked to make a self-assessment based on measurement items.

SEM has been used to study environmental behavior in a variety of fields including tourism [26], agriculture [27], occupational exposure [28], and risk perception [29]. One advantage of SEM is that one latent variable can be a dependent variable in one set of relationships and an independent variable in another set of relationships [30]. Because our hypothetical model involves multiple-path linkages that suggest complex associations among the variables, we chose SEM as an appropriate tool for this analysis. The equation for the SEM is presented below:

η= Bη+Γζ+ζ

where η is a vector composed of the endogenous latent variable; ξ is a vector composed of the exogenous latent variable; B represents the relations between the endogenous latent variables; Г is the influence of the exogenous latent variable on the endogenous variable; and ζ is the estimation error of the endogenous variable that cannot be explained completely.

## 4. Results

### 4.1. Data

One thousand questionnaires were distributed to residents in three communities in Guangzhou, China. These communities were pilot communities that do a relatively better job of waste separation. We randomly chose three or four residential buildings in each community to distribute the questionnaires. We placed envelopes containing the questionnaires and pens in the mailboxes of the residents and set up a questionnaire collection box in the residents’ building hall. The questionnaires were collected one week after they were distributed. The response rate was 24.6%, with 208 valid questionnaires, which was considered adequate for testing the stated hypotheses. In SEM, the sample size should not be too small or too large, because this type of an analysis relies on tests that are sensitive to the number of observations as well as to the magnitudes of differences in covariance matrices. Loehlin [31] recommends the use of at least 100 cases when examining more than 10 variables. The sample size should therefore be at least 30 more than eight times the number of latent variables. For this study, this assumption was valid for 208 cases and eight latent variables. The socio-demographic characteristics of the sample are summarized in Table 2.

**Table 2:** Summary of demographic characteristics.

Demographic Characteristics	Number of Cases	Percentage (%)
**Gender**		
Male	87	41.8
Female	121	58.2
**Age**		
<17	7	3.4
18–25	44	21.2
26–40	105	50.5
41–65	50	24.0
>66	2	1.0
**Education**		
Elementary school	8	3.8
Secondary school	25	12.0
University degree	130	62.5
Post graduate	45	21.6
**Occupation**		
Employee	108	51.9
Student	27	13.0
Retired	36	17.3
Housewife	7	3.4
Other	30	14.4
**Family monthly income(CNY)**		
<6000	77	37.0
6001–16,000	99	47.6
16,001–30,000	24	11.5
>30,001	8	3.8

### 4.2. The Measurement Model

To evaluate the measurement model, construct validity and composite reliability were examined (Table 3). Construct validity refers to whether an observed variable truly measures the construct as the researcher intends it to be measured. It was assessed based on factor analysis, composite reliability, and variance extracted. The factor loadings of all items in this study exceeded the recommended level of 0.6 [32]. Composite reliability reflects the internal consistency of a construct by indicating the extent to which the observed variables indicate the same unobserved concept. The construct reliability (CR) of the eight constructs exceeded the recommended level of 0.70 [33]. The average variance extracted (AVE), which reflects the overall amount of variance in the indicators accounted for by the latent construct, ranged between 0.543 and 0.865, exceeding the recommended level of 0.5 as suggested by Segars *et al.* [34]. In sum, the measurement model demonstrated adequate reliability and validity.

**Table 3:** The results of the measurement model assessment.

Construct	Mean	Factor Loading	CR ^a^	AVE ^b^
**Environment Knowledge (EK)**			0.936	0.790
EK1: I know how to properly separate the household waste	4.05	0.81		
EK2: Household separation can bring economic benefits	4.42	0.93		
EK3: Household separation can reduce environmental pollution	4.72	0.93		
EK4: Household separation can reduce the space occupied	4.51	0.88		
**Moral Obligation (MO)**			0.798	0.572
MO1: I separate waste out of my sense of responsibility to protect the environment	4.60	0.83		
MO2: Waste separation behavior is a virtue	4.57	0.81		
MO3: I would feel guilty if I didn’t separate waste properly	3.99	0.61		
**Attitude (A)**			0.898	0.745
A1: We have a responsibility to reduce the amount of waste generated	4.46	0.86		
A2: I feel angry if other people throw out waste incorrectly	4.55	0.87		
A3: Waste separation can create a better community environment	4.60	0.86		
**Subjective Norms (SN)**			0.879	0.708
SN1: My families expect me to separate waste	4.30	0.89		
SN2: My neighbors expect me to separate waste	3.86	0.77		
SN3: The community expects me to separate waste	4.15	0.86		
**Perceived Behavioral Control (PBC)**			0.850	0.655
PBC1: Whether I separate waste is dependent on me	3.94	0.83		
PBC2: I separate waste regardless of whether there are community incentives	4.17	0.87		
PBC3: Waste separation is a very easy thing for me	3.62	0.72		
**Separation Intention (I)**			0.951	0.865
I1: I am glad to engage in the government waste separation plan	4.26	0.93		
I2: I am glad to follow the guidance of the community	4.32	0.94		
I3: I am glad to continue to engage in a waste separation plan	4.35	0.92		
**Situational Factors (SF)**			0.776	0.543
SF1: I do not have time to separate my household waste	1.96	0.65		
SF2: There is not enough space for waste separation bins in my home	2.85	0.90		
SF3: Inconvenient waste separation facilities will influence my behavior	3.04	0.63		
**Separation Behavior (B)**			0.834	0.558
B1: I usually separate waste in accordance with the requirements of the community	4.03	0.76		
B2: I have insisted on waste separation for some time	3.79	0.74		
B3: I positively engage in waste separation	4.31	0.82		
B4: I always attempt to reduce the amount of waste in my home	4.35	0.66		

**^a^** CR = construct reliability; **^b^** AVE = average variance extracted.

The maximum likelihood (ML) method was used to test SEM. Measures of overall model fit included absolute, incremental, and parsimonious indices of fit. The best-known index of absolute fit is the Chi-square. While the Chi-square has been found to be sensitive to sample size, two indices were used to evaluate the overall absolute fit of the proposed model: the Goodness of Fit Index (GFI) and the Comparative Fit Index (CFI). To evaluate the fit of the proposed model and for incremental fit measures, the Adjusted Goodness of Fit Index (AGFI), the Incremental Fit Index (IFI), and the Normed Fit Index (NFI) were used. Finally, the Root Mean Square Error of Approximation (RMSEA) was used to evaluate the parsimonious fitness of the proposed model.

To assess the model fit, several indices were evaluated. These tests included Chi-square, GFI, RFI, NFI, IFI, TLI, CFI, and RMSEA. The suggested minimum acceptance values and the observed values from our model of these indices are presented in Table 4. The test of the overall model fit yielded χ^2^ (Chi-square) = 322.9 with 286 degrees of freedom and a *p*-value of more than 0.05. Thus, it is accepted that the model fits the data. The other indices are also higher than the suggested values. Overall, based on the recommended values gleaned from the literature, we conclude that the research model fitted the data quite well.

**Table 4:** Goodness-of-fit test results.

Fit index	Chi-Square (*p*-Value) [35]	GFI [35]	CFI [35]	RMSEA [36]	AGFI [37]	IFI [38]	NFI [39]
Suggested value	>0.05	>0.9	>0.9	<0.08	>0.8	>0.9	>0.9
Observed value	0.066	0.907	0.991	0.024	0.885	0.991	0.928
Conclusion	Accepted	Good fit	Good fit	Good fit	Good fit	Good fit	Good fit

### 4.3. The Structural Model

**Figure 3:**
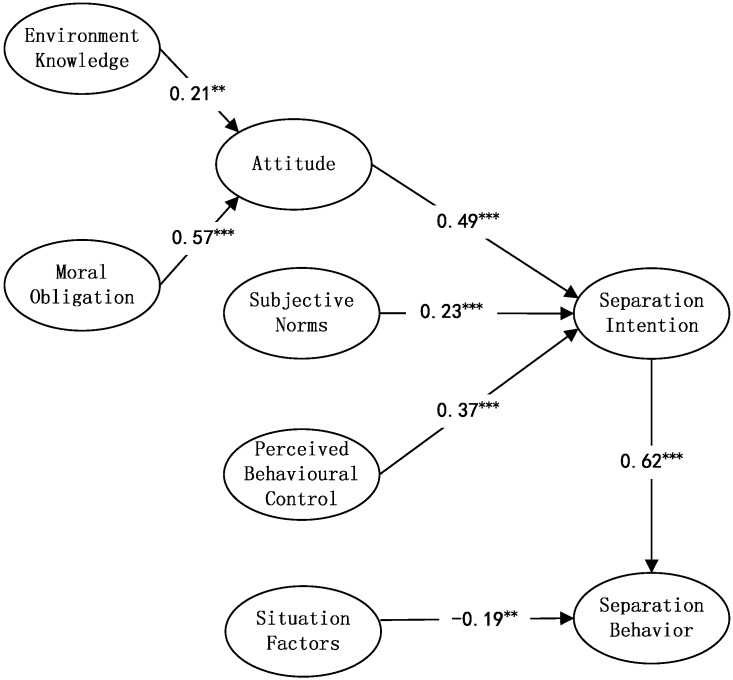
The estimated structural equation model. * Significant at *p* < 0.05; ** Significant at *p* < 0.01; *** Significant at *p* < 0.001.

The structural model was estimated using AMOS 17 software. Figure 3 illustrates the outcomes of the structured model with standardized parameters. The relationship between two predictors—*i.e.*, moral obligation as the independent variable, attitude as the mediator, and separation intention as the dependent variable—was determined by the proposed model. The results in Figure 3 support all the hypotheses.

### 4.4. Interpretation of the SEM Results

The results of the SEM analysis suggest that the attitude toward WSB had a positive influence on WSB intention, and this was the most significant effect. The separation behavior intention increased 0.49 units for each unit added to attitude, supporting H2. Attitude determined residents’ willingness to engage in waste separation behavior, evidencing a positive willingness based on a positive attitude, and waste separation-related knowledge and moral obligation significantly influenced the attitudes of the residents. Environmental knowledge and moral obligation both positively influenced attitude; however, moral obligation was the stronger for predicting attitude. Thus, H1a and H1b were supported. The results are consistent with those of previous studies [14,40], so strategies that emphasize individuals’ moral obligations are important for promoting household waste separation behaviors. Cultivation of residents’ responsibilities to protect the environment and promote the traditional virtues of the Chinese nation was particularly influential; the factor loadings were 0.83 and 0.81, respectively. It also helped residents to recognize that waste separation can be beneficial to the economy and that environmental protection will improve their attitude toward engaging in waste separation; the factor loadings for both were 9.3.

Subjective norms had a significant positive influence on separation behavior intention, but it was relatively weak. Separation behavior intention increased 0.23 units for each unit of increase in subjective norms, supporting H3. This reveals that residents’ social relations will likely promote or restrict their participation in waste separation, and families and communities have a greater impact on subjective norms. The factor loadings were 0.89 and 0.86, respectively, indicating that the studied community’s positive publicity and guidance will likely enhance residents’ subjective norms. Moreover, it is important to promote waste separation among young people in schools, thereby influencing their families’ subjective norms.

Perceived behavioral control had a significant positive effect on behavioral intentions. Separation behavior intention increases 0.37 units for each additional unit of perceived behavioral control, indicating that residents’ behavioral intentions were largely dependent on their self-control abilities. Self-control, in turn, will be enhanced when there is supervision and motivation in communities. Thus, H4 was supported. Because residents do not believe that waste separation is an easy task, the factor loading was only 0.72, so the communities are required to strengthen their guidance to make it easier to engage in waste separation, which will, in turn, improve residents’ perceived behavioral control.

Waste separation behavioral intentions had a significant positive influence on waste separation behavior; the path coefficient was 0.62, and H5 was supported. The factor loadings were all very high for items of intention—0.93, 0.94, and 0.92, respectively. These statistics indicate that increased intention of waste separation behavior can significantly improve the behavior, which is the focus for future waste separation work.

Situational factors and waste separation behavior were negatively correlated, and the influence was significant; thus, H6 was supported. Waste separation behavior will be restricted when residents do not have enough time and if space and facilities are inconvenient. It may be that the residents in our survey had enough time because the respondents were mostly housewives and retirees. Some residents believe that the existing waste delivery method is inconvenient, which relates to different collection and transportation methods in different communities. The proportion of respondents who find it inconvenient is higher in communities wherein residents are required to bring their own waste and place it in designated locations. Compared to the previous two items, a large proportion of the respondents agreed that there is not enough space to keep separate waste bins—the factor loading for this was 0.9. Therefore, space-intensive bins need to be designed.

### 4.5. Contributions of Demographic Values

One-way analysis of variance (ANOVA) was performed to determine whether demographic characteristics are important to the respondents’ views regarding waste separation behavior. The demographic characteristics are statistically significant in this regard when the *p*-value is less than 0.05. Table 5 shows the statistics of the effects of the demographic characteristics on the waste separation behavior variables. Gender, age, education, employment, and income do not appear to be statistically significant for any of the variables except separation behavior. Residents of different education levels have significantly different separation behaviors, with the undergraduate group demonstrating more positive behavior than the other groups.

**Table 5:** Analysis of variance of demographic variables.

Demographic Variable	Values of Significance ( *p*)					
	Attitude	Subjective Norm	Perceived Behavioral Control	Separation Intention	Situational Factors	Separation Behavior
**Gender**	0.700	0.465	0.780	0.784	0.634	0.232
**Age**	0.783	0.356	0.395	0.804	0.255	0.404
**Education**	0.899	0.475	0.573	0.909	0.733	0.043
**Employment**	0.668	0.976	0.706	0.869	0.690	0.657
**Income**	0.904	0.359	0.675	0.381	0.163	0.782

## 5. Discussion

We used the TPB theory as a framework for understanding household waste separation behaviors in Guangzhou, China. We added environmental knowledge, moral obligation, and situation factors to expand the TPB model. According to the results of this case study, the expanded TPB constructs were significant predictors of waste separation behavior. Attitude was the strongest predictor of waste separation behaviors in our study, with the largest path coefficient of all the TPB constructs in our model.

A preliminary conclusion of this study is that the expanded TPB is a good model to measure WSB and, together with SEM, we can clearly reveal the quantitative relationships between observable variables and latent variables and between the latent variables as well. The study shows that moral obligation plays the largest role in determining individuals’ attitude. This finding is consistent with those of previous studies [7,40], suggesting that strategies that emphasize individuals’ moral motivations are important in promoting household waste separation behaviors. Cultivating residents’ responsibility to protecting the environment and promoting traditional virtues of the Chinese nation would be particularly effective.

Guangzhou implemented the “Guangzhou Solid Waste Separation Management Interim Provisions” in April 2011, making it the first city in China to formulate local regulations for waste separation. These guidelines were revised and finalized in 2013. In Guangzhou, the Guangzhou City Management Committee (GCMC) mostly manages waste separation. However, in fact, many other departments are also involved, such as Environmental Protection, Education, Planning, Traffic, and Finance. Problems faced in implementation relate to management, limited impact, and low public participation.

With regard to low participation rates, the rate for this survey was 24.6%, which is higher than usual for postal mail surveys [12], which may be attributed to our different data collection method. We put envelopes containing the questionnaire in the doorways of residents and in recycling bins in the apartment lobby. Though the participation rate met our expectations, it also demonstrates the low participation rate among the general population.

Public participation may be improved with campaigns that emphasize individuals’ moral obligations to separate household waste. Such campaigns should aim to improve individuals’ environmental knowledge and individuals’ waste separation abilities (perceived behavioral control). These campaigns should target a combination of government, schools, and residents. It is the government’s responsibility to formulate and implement relevant laws and regulations and to take action to implement these in numerous communities. Schools can initiate many environmental activities in the community through their students and these activities can enhance residents’ awareness of waste separation as well as their passion for practical action. Residents can develop incentives to encourage other residents to be active in separating their waste, which would not happen without residents’ active participation.

Generally, respondents with different demographic characteristics were not significantly different in WSB. This could be related to the bias in our sample, which comprised mostly housewives and retirees. Respondents between the ages of 26–40 accounted for 50.5% of the total, and they were mostly housewives. This is consistent with the actual situation in cities—particularly in large cities—where the husband is the primary breadwinner while the wife is responsible for childrearing. Because of this, household waste separation is performed by wives and retirees. This finding indicates that housewives and retirees should be the key target for increasing engagement in environmental moral education. The Average Wage of Fully Employed Staff and Workers in Urban Units in Guangzhou City in 2013 was CNY 69,692, which means that the per capita monthly income was about CNY 5800; these results match our samples.

Although our TPB constructs significantly predicted waste separation behaviors, some limitations remain in our study. First, we used self-report as a proxy for actual behavior. It is possible that we overestimated participation in household waste separation behaviors because declared behavior does not always reflect actual behavior and may thus lead to overestimation (particularly of morally desirable behavior). Second, the samples in our study are biased. According to the sixth national census of Guangzhou City, conducted in 2010, 22.5% of the residents have university degrees, whereas the percentage of our respondents with degrees was 84%. This may be because the three pilot communities where we conducted the survey are places in which the urban elite tends to live. Finally, our sample size was not large enough; for this reason, we could not examine the deep impact of different socioeconomic factors on the residents’ WSB. In the future, we will expand the sample size to reveal the larger impact of these factors on residents’ WSB.

## 6. Conclusions

Based on questionnaire data from residents’ separation behavior, we found that attitude, subjective norms, perceived behavioral control, intention, and situational factors significantly predicted household waste behaviors in Guangzhou, China. In the future, policies and campaigns should focus on evoking residents’ active participation in waste separation, strengthening the propaganda of environmental knowledge, educating residents in terms of moral obligation, and focusing on helping residents to establish a sense of responsibility for protecting the environment.

Situational factors had a significant negative impact on waste separation behavior, indicating that lack of time and inconvenience in terms of place will likely inhibit residents’ waste separation behavior. The results of our survey implied that, although residents felt that they had enough time to participate in waste separation, its level of convenience should be enhanced.

Furthermore, the socioeconomic attributes of residents had an impact on waste separation behavior. For example, the residents’ educational level has the greatest influence on waste separation behavior. Residents of different educational levels exhibited different separation behaviors, with undergraduates demonstrating more positive behavior.

One reason for the continued growth of Guangzhou municipal solid waste is the low percentage of residents who participate in waste separation. The communities we chose are those that have done a good job in waste separation, but the questionnaire response rate was 24.6%. Future studies should focus on designing and measuring the effectiveness of public campaigns concerning waste separation behavior. Considering that cities in China appear to be experiencing a landfill overload, waste management is becoming increasingly difficult; thus, measures for increasing the resident participation rate and reducing the amount of waste to be treated are the most important for China to address.
